# The Dietary Constituent Falcarindiol Promotes Cholesterol Efflux from THP-1 Macrophages by Increasing ABCA1 Gene Transcription and Protein Stability

**DOI:** 10.3389/fphar.2017.00596

**Published:** 2017-09-01

**Authors:** Limei Wang, Veronika Palme, Nicole Schilcher, Angela Ladurner, Elke H. Heiss, Herbert Stangl, Rudolf Bauer, Verena M. Dirsch, Atanas G. Atanasov

**Affiliations:** ^1^Department of Pharmacognosy, University of Vienna Vienna, Austria; ^2^Department of Pharmacology, School of Pharmacy, Qingdao University Qingdao, China; ^3^Center for Pathobiochemistry and Genetics, Institute of Medical Chemistry, Medical University of Vienna Vienna, Austria; ^4^Department of Pharmacognosy, Institute of Pharmaceutical Sciences, Karl-Franzens-University Graz Graz, Austria; ^5^Department of Molecular Biology, Institute of Genetics and Animal Breeding of the Polish Academy of Sciences Lesznowola, Poland

**Keywords:** falcarindiol, cholesterol efflux, ABCA1, protein degradation, PPARγ

## Abstract

We report increased cholesterol efflux from macrophages in the presence of falcarindiol, an important dietary constituent present in commonly used vegetables and medicinal plants. Falcarindiol (3–20 μM) increased cholesterol efflux from THP-1-derived macrophages. Western blot analysis showed an increased protein level of ABCA1 upon falcarindiol exposure. Quantitative real-time PCR revealed that also ABCA1 mRNA level rise with falcarindiol (10 μM) treatment. The effect of falcarindiol on ABCA1 protein as well as mRNA level were counteracted by co-treatment with BADGE, an antagonist of PPARγ. Furthermore, falcarindiol significantly inhibited ABCA1 protein degradation in the presence of cycloheximide. This post-translational regulation of ABCA1 by falcarindiol occurs most likely by inhibition of lysosomal cathepsins, resulting in decreased proteolysis and extended protein half-life of ABCA1. Taken together, falcarindiol increases ABCA1 protein level by two complementary mechanisms, i.e., promoting ABCA1 gene expression and inhibiting ABCA1 protein degradation, which lead to enhanced cholesterol efflux.

## Introduction

Cardiovascular disease (CVD) associated with atherosclerosis is a leading cause of mortality in the world. Cholesterol metabolism has been considered as a major player in the pathogenesis of CVD. Excessive cholesterol deposition resulting in plaques is a critical cause of atherosclerosis. Reverse cholesterol transport (RTC) is a complex multi-step process overall resulting in the relocation of cholesterol from peripheral tissues to the liver for excretion into the bile and ultimately the feces. Cholesterol efflux, the initial step of RTC, is known to enhance the export of excessive cholesterol from peripheral tissues, in particular macrophages, and thereby to inhibit the accumulation of cholesterol in the wall of arteries (Fielding and Fielding, 1995). There is strong evidence that the cholesterol efflux capacity is inversely associated with the incidence of CVD (Rohatgi et al., 2014). Therefore, the enhancement of cholesterol efflux from macrophages might prevent CVD, making it a potential strategy for pharmacological interventions. Key determinant of cholesterol efflux is the expression level and activity of transmembrane transporter proteins such as the ATP-binding cassette transporter A1 (ABCA1) and G1 (ABCG1) and the scavenger receptor class B member 1 (SR-B1) (Duffy and Rader, 2006). Among them, ABCA1 is the predominant one and it contributes to most of the cholesterol transport in macrophages (Rosenson et al., 2012; Du et al., 2015). ABCA1 mediates mostly transfer of cholesterol to lipid–poor apolipoprotein A1 (apo A1), but it can also mediate cholesterol transport using high density lipoproteins (HDL) as an acceptor (Du et al., 2015). Modulation of the ABCA1 protein level is considered an important regulator for cholesterol efflux. In general, the level of ABCA1 protein is determined by *de novo* synthesis and protein degradation. Several nuclear receptors are implicated in the regulation of ABCA1 gene transcription. Thus, the activation of the ligand-dependent nuclear receptors peroxisome proliferator-activated receptor gamma (PPARγ) and subsequently the liver X receptor (LXR), can lead to increased ABCA1 gene transcription (Chawla et al., 2001; Chinetti et al., 2001; Ozasa et al., 2011). The ABCA1 protein half-life is largely regulated via the proteolytic activity of calpain-, lysosome-, and ubiquitin-degradation pathways (Wang et al., 2003; Mizuno et al., 2011; Liu and Tang, 2012; Yokoyama et al., 2012). Cathepsins represent key lysosomal proteases (Turk et al., 2012) and diverse cathepsin isoforms (e.g., cathepsin B, D, K, L, S) are known to be expressed in macrophages (Punturieri et al., 2000; Beers et al., 2003; Bracke et al., 2005; Vasiljeva et al., 2006).

**Graphical Abstract:**
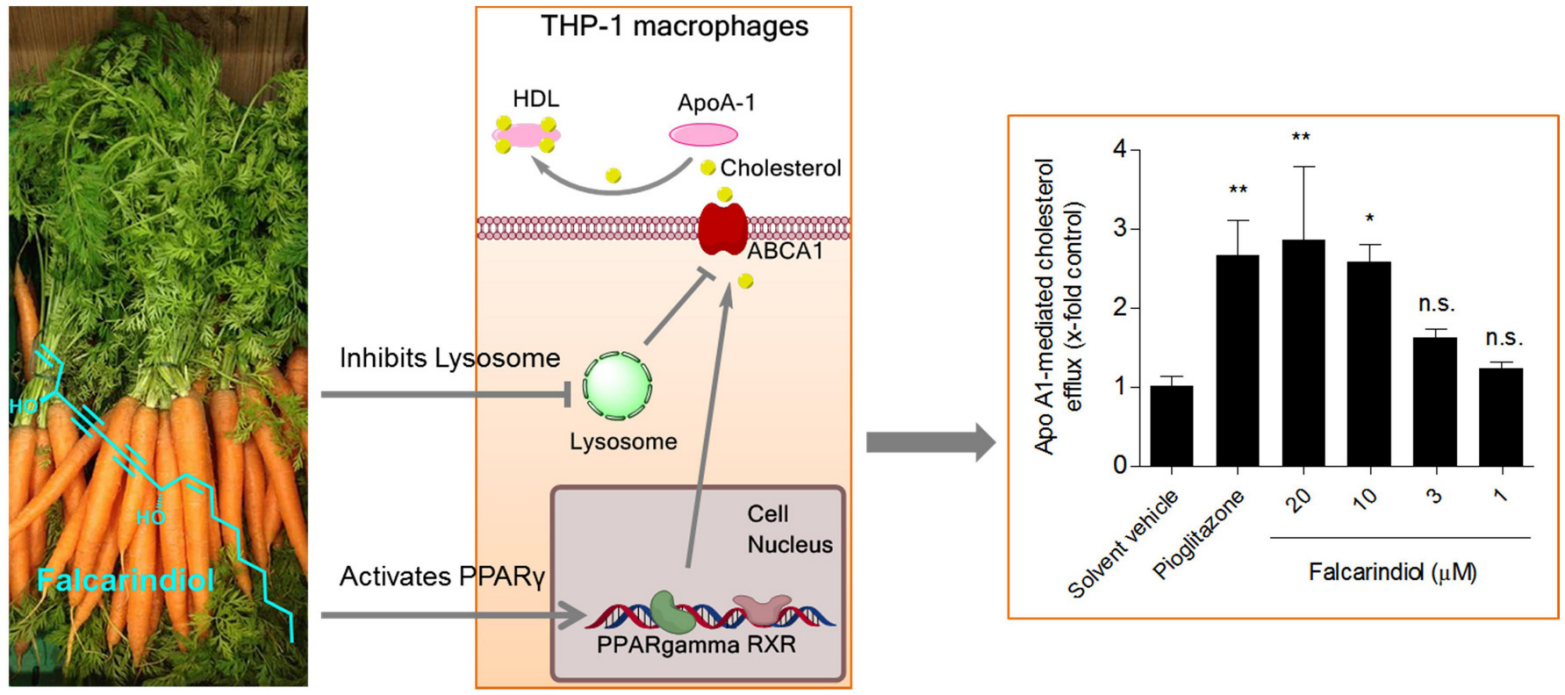
The dietary constituent falcarindiol promotes macrophage cholesterol efflux by increasing ABCA1 transporter gene transcription (via PPARγ activation) and protein stability (via inhibition of lysosomal proteolysis).

**Figure 1:**
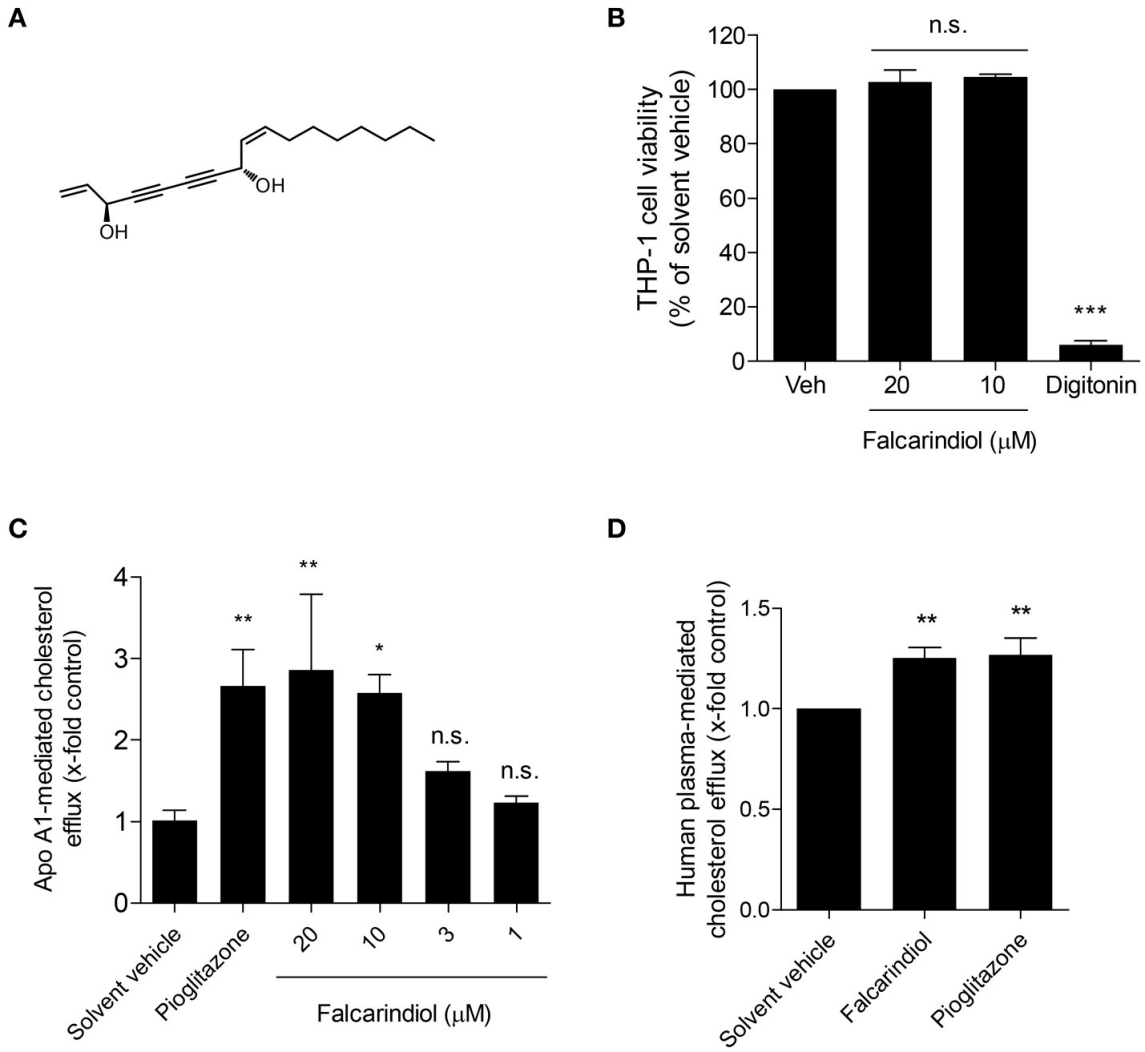
Chemical structure of falcarindiol **(A)**, its effect on cell viability **(B)**, and cholesterol efflux in THP-1-derived macrophages **(C,D)**. **(B)** Differentiated THP-1 macrophages were incubated with solvent vehicle control (DMSO), 50 μg/mL digitonin, or the indicated concentrations (10–20 μM) of falcarindiol. After 24 h, the increased fluorescent signal from resazurin conversion was detected and calculated as the percentage of cell viability. **(C,D)** THP-1-derived macrophages were loaded with [^3^H]-cholesterol together with the indicated treatments for 24 h. On the next day, macrophages were washed twice with pre-warmed PBS and incubated with the same compounds [solvent vehicle (DMSO), falcarindiol (1–20 μM for apo A1-mediated cholesterol efflux, or 10 μM for human plasma-mediated cholesterol efflux), or the PPARγ agonist pioglitazone (10 μM) as positive control] with or without 10 μg/mL apo A1 **(C)** or 1% human plasma **(D)** dissolved in serum-free medium for 6 h. Extracellular as well as intracellular radioactivity were quantified with a scintillation counter. All values are means ± *SD* (*n* = 3) and vs. solvent vehicle control (DMSO): ^*^p < 0.05, ^**^p < 0.01, ^***^p < 0.001, n.s, not significant (ANOVA/Bonferroni).

Natural products have proven to be an excellent source of molecules with promising pharmacological activities and novel mechanisms of action (Atanasov et al., 2015). Here, we report the identification and characterization of the natural product falcarindiol (Figure 1A) as a novel enhancer of macrophage cholesterol efflux. Falcarindiol is a C(17)-polyacetylene and a typical constituent of roots and rhizomes of Apiaceae plants used worldwide, including in Europe, such as the commonly used vegetables and herbs carrot (*Daucus carota* L.) (Garrod et al., 1978), celery (*Apium graveolens* L.) (Zidorn et al., 2005), *Notopterygium incisum* K.C.Ting ex H.T.Chang (Zschocke et al., 1997), and *Aegopodium podagraria* L. (Kemp, 1978), among others. Taking the important role of PPARγ in the regulation of macrophage cholesterol efflux into consideration, we have chosen this natural product as a study subject in the context of cholesterol efflux because falcarindiol, extracted from the rhizomes and roots of *Notopterygium incisum* K.C.Ting ex H.T.Chang, was recently identified as a partial PPARγ agonist in HEK-293 cells transfected with a PPARγ-responsive luciferase reporter (Atanasov et al., 2013).

## Materials and methods

### Chemical reagents

Roswell Park Memorial Institute 1640 (RPMI-1640) medium was obtained from Lonza (Basel, Switzerland). Fetal bovine serum (FBS) was purchased from Gibco via Invitrogen (Lofer, Austria). [^3^H]-Cholesterol was bought from Perkin Elmer Life Sciences (Vienna, Austria) and pioglitazone was from Molekula (Munich, Germany). Albumin faction V, fatty acid free BSA was obtained from Carl Roth (Graz, Austria). Bisphenol A diglycidyl ether (BADGE), resazurin sodium salt, water soluble unesterified cholesterol, apolipoprotein (apo) A1, phorbol 12-myristate 13-acetate (PMA), lactacystin, calpeptin, chloroquine, and cycloheximide were provided by Sigma-Aldrich (Vienna, Austria). Falcarindiol was isolated from the roots and rhizomes of the traditional Chinese herbal drug Radix Notopterygii, with a purity over 95% as determined by High Performance Liquid Chromatography (HPLC) (Zschocke et al., 1997; Liu et al., 2014). Cathepsin B, L, D, and K Activity Fluorometric kits were purchased from BioVision via THP Medical Products GmbH (Vienna, Austria). Cathepsin S activity assay kit (fluorometric) was bought from Abcam (Cambridge, UK). Antibodies were obtained from the following companies: ABCA1, ABCG1, SR-B1 from Novus Biologicals via THP Medical Products GmbH (Vienna, Austria), anti-actin antibody from MP biologicals (Illkirch, France), horseradish peroxidase-conjugated goat anti-rabbit secondary antibody from New England Biolabs (Frankfurt, Germany), horseradish peroxidase conjugated goat anti-mouse secondary antibody from Upstate (Millipore, Vienna, Austria).

### Cell culture

Human monocytic THP-1 cells were acquired from ATCC® and grown in suspension in RPMI-1640 medium supplemented with 2 mM glutamine, 100 U/mL benzylpenicillin, 100 mg/mL streptomycin, and 10% FBS in T175 flasks. For experiments, differentiation was induced by seeding THP-1 monocytes in 96-, 24-, or 6- well plates under treatment of 200 nM PMA for 72 h (seeding cell density of 0.2 × 10^6^ cells per mL). All of the tested compounds were dissolved in dimethyl sulfoxide (DMSO) and stored at −20°C. Control cells were always treated with an equal volume of solvent (DMSO) and its final concentrations did not exceed 0.1%.

### Cholesterol efflux assay

The cholesterol efflux assay (Wang et al., 2015) was adapted from previous studies (Chawla et al., 2001; Chinetti et al., 2001). Briefly, THP-1-derived macrophages were incubated for 24 h with the compounds at the indicated concentrations [solvent vehicle (DMSO), falcarindiol (1–20 μM), the PPARγ agonist pioglitazone (10 μM) as positive control] together with [^3^H]-cholesterol in serum free medium supplemented with 0.1% BSA and 10 μg/mL unesterified cholesterol. On the next day, cells were washed twice with pre-warmed PBS and incubated with the same compounds with or without 10 μg/mL apo A1 or 1% human plasma dissolved in serum-free medium for 6 h. Extracellular as well as intracellular radioactivity were quantified with scintillation counting. The apo A1- as well as human plasma-mediated cholesterol efflux was calculated as follows:

$$\begin{array}{lll} Apo\;A1\;mediated\;cholesterol\;efflux\;\% & = & \left(\frac{\left(extracellular\;cpm\right)\;apo\;A1}{\left(total\;cpm\right)\;apo\;A1}-\frac{\left(extracellular\;cpm\right)\;no\;apo\;A1}{\left(total\;cpm\right)\;no\;apo\;A1}\right)\times 100\\ Human\;plasma\;mediated\;cholesterol\;efflux\;\% & = & \left(\frac{\left(extracellular\;cpm\right)\;plasma}{\left(total\;cpm\right)\;plasma}-\frac{\left(extracellular\;cpm\right)\;no\;plasma}{\left(total\;cpm\right)\;no\;plasma}\right)\times 100 \end{array}$$

### Resazurin conversion assay

The viability of THP-1-derived macrophages was assessed by resazurin conversion. The assay was performed as previously described (Wang et al., 2015) by quantification of the conversion of the low fluorescent resazurin to high fluorescent resorufin.

### Gel electrophoresis and immunoblot analysis

THP-1 cells were seeded in 6-well plates and differentiated into macrophages with 200 nM PMA for 72 h. The macrophages were treated with the indicated compounds at the corresponding time points, as described in detail in each figure legend. Cellular proteins were extracted using NP40 buffer (150 mM NaCl; 50 mM HEPES (pH 7.4); 1% NP40; 1% protease inhibitor Complete™ (Roche); 1% phenylmethylsulfonyl fluoride (PMSF); 0.5% Na_3_VO_4_; 0.5% NaF) and quantified with the Bradford method. For western blot analysis, an equal amount of protein (20 μg) was separated via SDS-PAGE and transferred onto a PVDF membrane. The membrane was blocked for 1 h at room temperature with 5% non-fat milk, and then incubated at 4°C overnight with primary polyclonal antibody against ABCA1, ABCG1 or SR-B1 (1:500) or actin (1:10,000). After being washed for three times (15 min each time), the membranes were incubated with a secondary antibody [horseradish peroxidase-conjugated goat anti-rabbit (1:500) or anti-mouse (1:10,000)] for 1 h at room temperature. The signals were detected and visualized using a LAS-3000 luminescent image analyzer (Fujifilm, Düsseldorf, Germany) using AIDA image analyzer 4.06 software (Raytest, Sprockhövel, Germany). The indicated protein band intensity was normalized to that of actin.

### RNA extraction and analysis

THP-1 cells were seeded in 6-well plates and differentiated into macrophages with a 200 nM PMA treatment for 72 h. The macrophages were treated with the indicated compounds at the indicated time points, which are described in detail in the figure legends. Total cellular RNA was extracted from the treated THP-1 macrophages using peqGOLD Total RNA kits (PeqLab, Linz, Austria) according to the manufacturer's instructions. One microgram of total RNA was used for cDNA synthesis according to the protocol of the High Capacity cDNA Reverse Transcription Kit together with RNase Inhibitor (Applied Biosystems). LightCycler® 480 SYBR Green I Master kit (Roche) was used for quantitative real-time PCR (qRT-PCR) with 40 ng cDNA from each sample for triplicate measurements. Amplification cycles were detected using the LightCycler 480 system (Roche). ABCA1 (HS_ABCA1_1_SG QuantiTect primer assay, Cat.no.: #QT00064869, QIAGEN) and 18S (Hs_RRN18S_1_SG QuantiTect Primer assay, Cat.no.: #QT00199367, QIAGEN) primers were used, and the quantification was performed with the Δ*C*_*T*_ method.

### Determination of cathepsin activity

The cathepsin activity assays were performed according to the manufacturer's instructions. In general, THP-1-derived macrophages were cultured for 24 h with solvent control (DMSO) or falcarindiol (10 μM). After incubation, cells were washed once with cold PBS and lysed with chilled cell lysis buffer. Protein concentration was determined using the Bradford method. Hundred micrograms of protein per reaction in 50 μL of cell lysis buffer was used for the activity measurement. Fifty microliters of reaction buffer and 2 μL of the respective cathepsin substrate were added to 50 μL cell lysates. The assay plate was incubated at 37°C for 1 h protected from light. The fluorescence was quantified with Tecan Infinite 200® Pro plate reader (Vienna, Austria).

### Statistical methods

Statistical analysis was performed with data acquired from at least three independent experiments using GraphPad Prism software version 4.03 (GraphPad Software Inc., La Jolla, CA, USA). Figures with bar graphs represent mean ± *SD*. To determine statistical significance, one- or two- way analysis of variance (ANOVA) were performed and p < *0.05* was considered significant.

## Results

### Falcarindiol promotes cholesterol efflux in Thp-1-derived macrophages

In a previous study, we showed that falcarindiol activates PPARγ in HEK-293 cells transfected with a PPARγ-responsive luciferase reporter (Atanasov et al., 2013). Considering that PPARγ is a known regulator of cholesterol efflux (Chawla et al., 2001; Chinetti et al., 2001), we aimed to investigate whether falcarindiol can promote cholesterol efflux in THP-1-derived macrophages. Since apo A1, the nascent and lipid free form of HDL, is the most significant acceptor for cholesterol effluxed from macrophages (Du et al., 2015), we first studied cholesterol efflux mediated by apo A1. Falcarindiol enhanced apo A1-mediated cholesterol efflux in a concentration-dependent manner when applied at 1–20 μM (without effect on cell viability; Figure 1B) with a half maximal effective concentration (EC_50_) of 5.8 μM (Figure 1C). Moreover, 10 μM falcarindiol also significantly enhanced 1% human plasma-mediated cholesterol efflux (Figure 1D) as plasma containing HDL particles served as cholesterol acceptors in the process of cholesterol efflux. Pioglitazone, a PPARγ agonist known to upregulate cholesterol efflux (Ozasa et al., 2011), was used as positive control both in presence of apo A1 and human plasma (Figures 1C,D).

### Falcarindiol enhances ABCA1 protein expression

**Figure 2:**
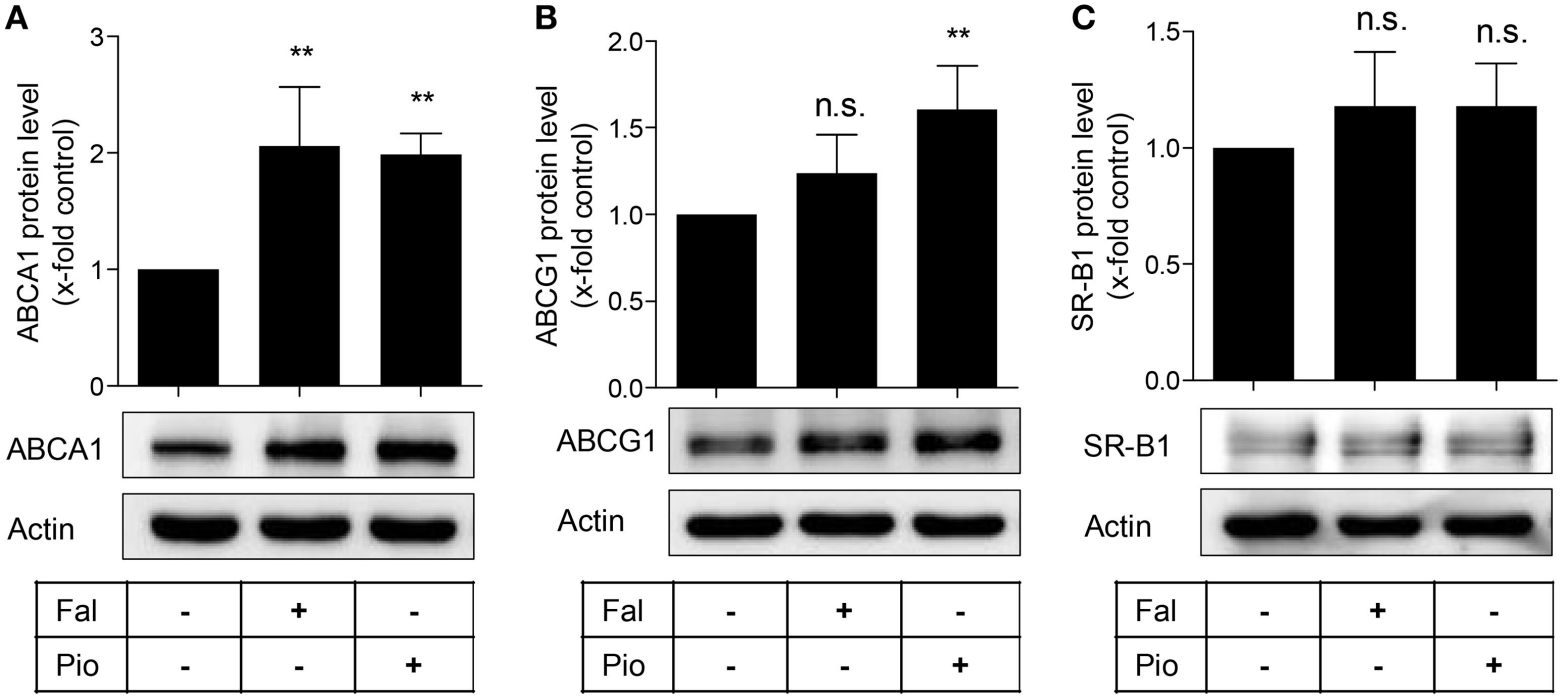
Falcarindiol increases ABCA1 protein level. Differentiated THP-1 macrophages were treated with solvent vehicle control (DMSO), falcarindiol (Fal, 10 μM), or the PPARγ agonist pioglitazone (Pio, 10 μM). After 24 h, cells were lysed and 20 μg protein was resolved via SDS-PAGE. Immunoblotting was performed with antibodies against the indicated proteins, ABCA1 **(A)**, ABCG1 **(B)**, and SR-B1 **(C)** as well as actin as loading control. All experiments were performed four times and values are presented as means ± *SD* and vs. solvent vehicle control (DMSO): ^**^p < 0.01, n.s. not significant (ANOVA/Bonferroni).

**Figure 3:**
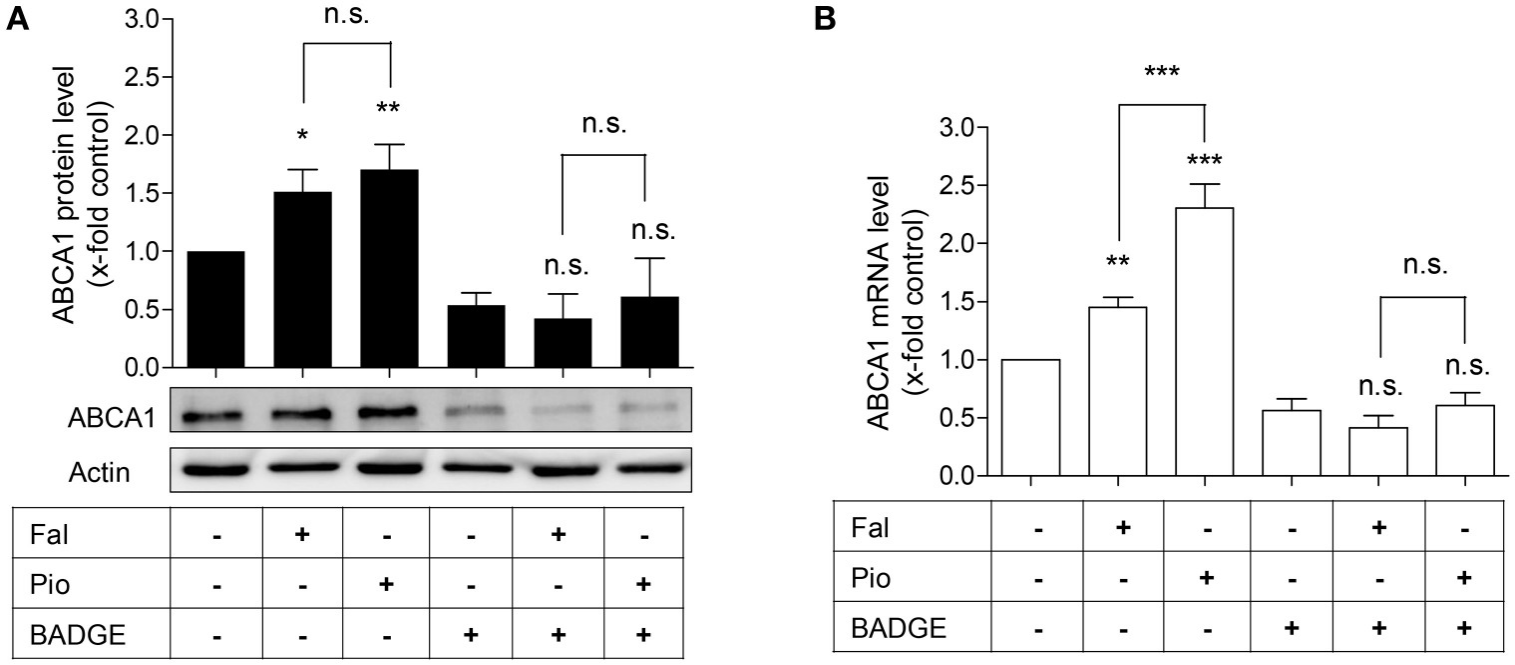
The PPARγ antagonist, BADGE, blocks falcarindiol-mediated ABCA1 protein and mRNA induction. Differentiated THP-1 macrophages were incubated for 24 h with falcarindiol (Fal, 10 μM) or pioglitazone (Pio, 10 μM) with or without 50 μM BADGE. ABCA1 protein **(A)** and mRNA **(B)** level were analyzed by western blotting and qPCR, respectively. All data are mean ± *SD* (*n* = 3), ^*^p < 0.05, ^**^p < 0.01, ^***^p < 0.001, n.s. not significant vs. solvent vehicle control (DMSO) (ANOVA/Bonferroni).

The transmembrane proteins ABCA1, ABCG1, and SR-B1 are transporters mediating cholesterol efflux (Duffy and Rader, 2006). Therefore, we investigated protein expression of these three transporters upon treatment with falcarindiol in THP-1-derived macrophages. As evident in Figure 2A, ABCA1 protein level significantly increased in response to falcarindiol (10 μM). At the same time, there was no significant upregulation of ABCG1 (Figure 2B) or the bi-directional transporter SR-B1 (Figure 2C; Stangl et al., 1999). Next, we investigated whether increased ABCA1 protein level is associated with an increase in ABCA1 mRNA level. ABCA1 mRNA level indeed significantly increased upon falcarindiol (10 μM) treatment (Figure 3B, left).

In macrophages, enhanced ABCA1 mRNA level might result from PPARγ and subsequent LXR activation (Chawla et al., 2001; Chinetti et al., 2001). We, therefore, asked whether the increased ABCA1 mRNA level induced by falcarindiol might be due to PPARγ-activation. To check this possibility, we used the PPARγ antagonist, BADGE (50 μM), to block the activity of PPARγ in THP-1-derived macrophages (Hinz et al., 2003). Indeed, BADGE blocked the falcarindiol-mediated increase of ABCA1 protein as well as mRNA level (Figures 3A,B).

### Falcarindiol inhibits ABCA1 protein degradation

**Figure 4:**
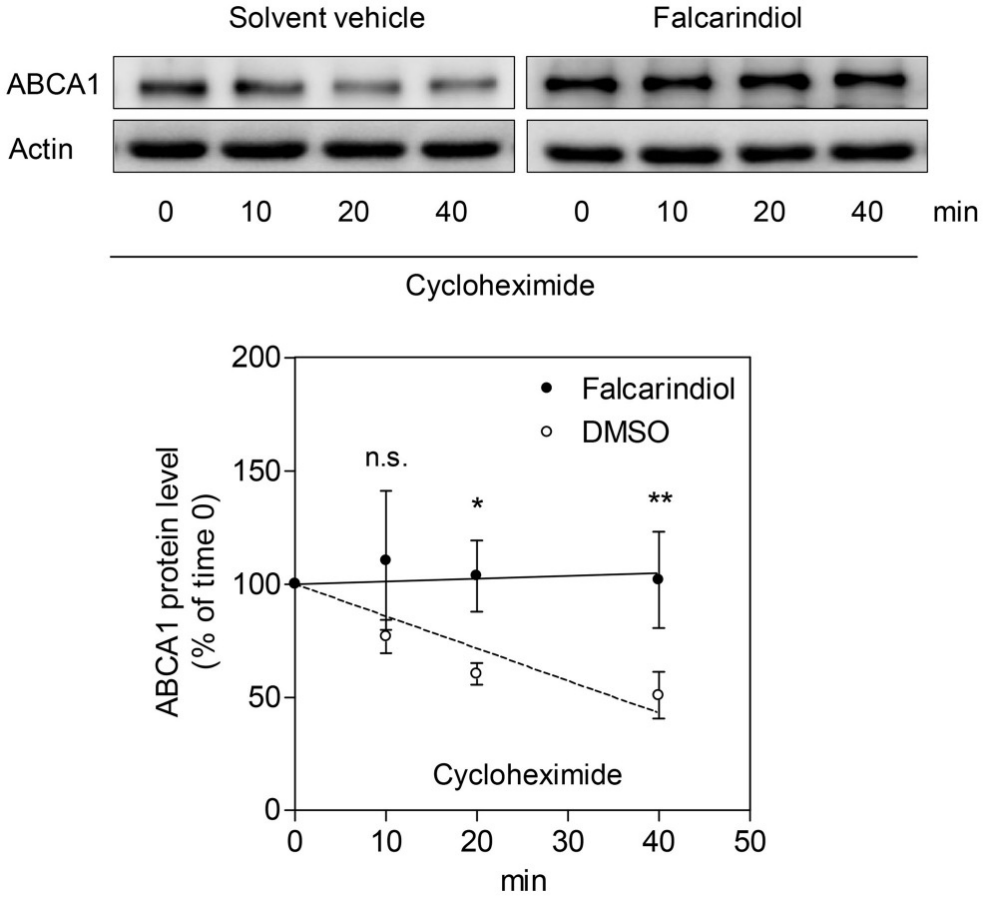
Falcarindiol inhibits ABCA1 protein degradation. Differentiated THP-1 macrophages were stimulated with 10 μM falcarindiol (black circles) or an equal amount of the solvent vehicle control (DMSO; white circles). After 24 h incubation, cells were treated with cycloheximide (140 μM) and lysed at different time points (0, 10, 20, 40 min). Western blotting was used to monitor the decline of ABCA1 protein level upon cycloheximide treatment in the presence or absence of falcarindiol. All data are mean ± *SD* (*n* = 3) and vs. solvent vehicle control (DMSO): ^*^p < 0.05, ^**^p < 0.01, n.s. not significant (two-way ANOVA/Bonferroni).

Interestingly, the ABCA1 mRNA up-regulation by falcarindiol was significantly lower than the mRNA induction caused by the PPARγ agonist pioglitazone (Figure 3B, on the left), while the induction of ABCA1 protein level by falcarindiol and pioglitazone was equally potent (Figure 3A, on the left). This inconsistency indicated that an additional effect of falcarindiol might exist, which could enhance ABCA1 protein level besides the increase of ABCA1 mRNA level. To explore this possibility, we measured the rate of ABCA1 protein degradation in the presence and absence of falcarindiol. THP-1-derived macrophages were incubated with falcarindiol or solvent vehicle (DMSO) for 24 h and were then further treated with cycloheximide (140 μM) to block *de novo* protein synthesis (Arakawa et al., 2009). As evident in Figure 4, ABCA1 protein degradation was significantly inhibited in the presence of falcarindiol showing that the compound interferes with the ABCA1 protein turnover rate.

### Falcarindiol inhibits lysosomal cathepsin-dependent ABCA1 degradation

**Figure 5:**
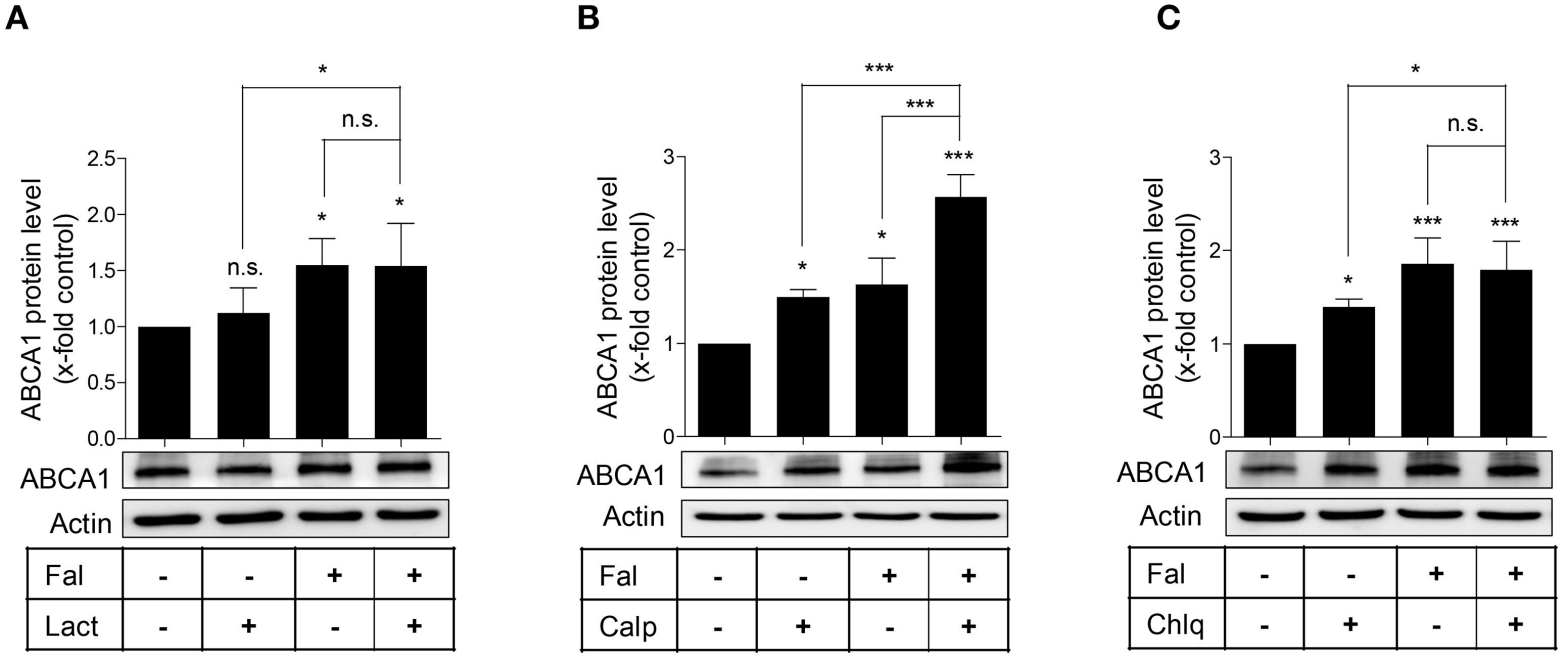
Effect of specific inhibitors of the proteasome **(A)** calpain **(B)**, and lysosomal **(C)** protein degradation pathways on ABCA1 protein level in the presence or absence of falcarindiol. Differentiated THP-1 macrophages were pre-treated with or without falcarindiol (Fal) at 10 μM for 24 h and incubated for another 3 h with the proteasome inhibitor lactacystin (Lact) at 10 μM **(A)**, the calpain inhibitor calpeptin (Calp) at 30 μg/mL **(B)**, or the lysosomal inhibitor chloroquine (Chlq) at 100 μM **(C)**. Cells were lysed and protein was resolved via SDS-PAGE. Western blot analysis was used to monitor ABCA1 protein level under the indicated treatment conditions. All data are mean ± *SD* (*n* = 4) and vs. solvent vehicle control (DMSO): ^*^p < 0.05, ^***^p < 0.001, n.s. not significant (one-way ANOVA/Bonferroni).

To further explore how falcarindiol inhibits ABCA1 protein degradation, we first investigated, which protein degradation pathway could be affected by falcarindiol in THP-1-derived macrophages. As presented in Figure 5A, the application of lactacystin, an ubiquitin proteasome inhibitor, failed to affect ABCA1 protein level in our experimental setting. However, the application of calpeptin (a calpain inhibitor) and chloroquine (a lysosome inhibitor) (Figures 5B,C) increased ABCA1 protein level, similarly to falcarindiol. Falcarindiol had an additive effect on ABCA1 protein level upon co-treatment with calpeptin, indicating that its mechanism of action is likely different from the calpain inhibitor calpeptin. However, there was no difference observed with or without chloroquine in the presence of falcarindiol, suggesting that falcarindiol might interfere with protein degradation in the lysosome, mimicking the effect of chloroquine (Figure 5C).

**Figure 6:**
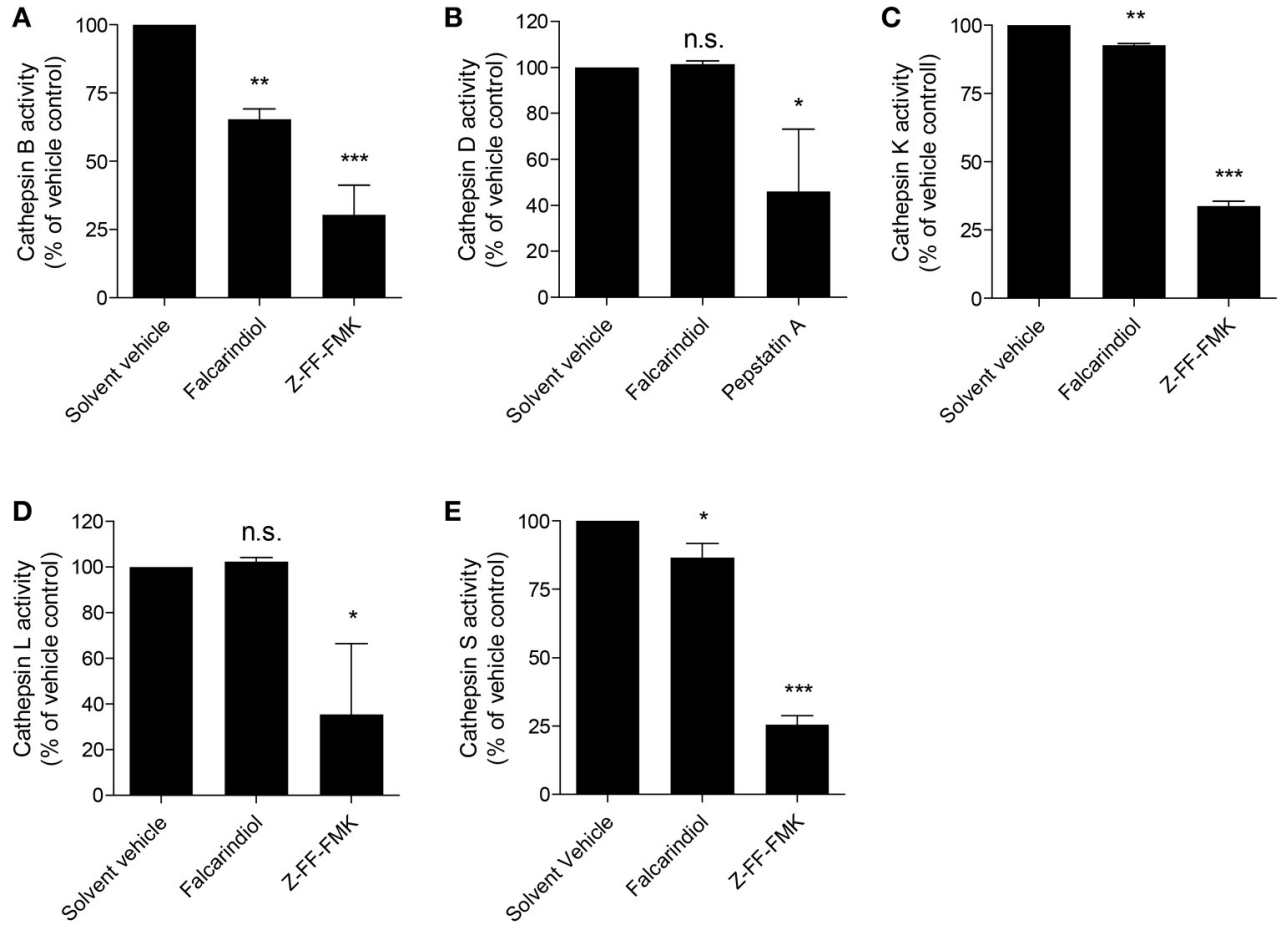
Falcarindiol inhibits several lysosomal cathepsins. **(A–E)** Differentiated THP-1 macrophages were incubated for 24 h with or without falcarindiol (10 μM). Cells were lysed and cathepsin activity (S, B, L, K, and D) was determined by fluorescence measurements. Irreversible inhibitors of cathepsin B, S, L, and K (Z-FF-FMK; 1 μM) and cathepsin D (pepstatin A; 10 μM) were used as positive controls and directly added to the control lysates. All data are mean ± *SD* (*n* = 3) and vs. solvent vehicle control (DMSO): ^*^p < 0.05, ^**^p < 0.01, ^***^p < 0.001, n.s. not significant (one way-ANOVA/Bonferroni).

The lysosomal degradation pathway is a well-known regulator of ABCA1 protein turnover (Neufeld et al., 2001; Santamarina-Fojo et al., 2001; Mizuno et al., 2011) and cathepsins are well established proteases mediating the lysosomal proteolysis in general (Turk et al., 2012; Appelqvist et al., 2013). Therefore, we investigated whether the proteolytic activity of lysosomal cathepsins is affected by falcarindiol. Figures 6A–E shows that among the tested cathepsins (B, D, K, L, S) the activity of cathepsin B, S, and K were significantly suppressed by 35, 14, and 8%, respectively. Overall, these data indicate that falcarindiol leads to the suppression of ABCA1 degradation and inhibits lysosomal cathepsins.

## Discussion

In this study, we describe for the first time that falcarindiol, a typical constituent of Apiaceae vegetables, stimulates cholesterol efflux by promoting ABCA1 gene expression in THP-1-derived macrophages. In addition, we show that falcarindiol increases ABCA1 protein stability and inhibits the proteolytic activity of lysosomal cathepsins.

Macrophage cholesterol efflux is a process counteracting the transformation of macrophages into pro-atherogenic foam cells. Thus, the study of this process and molecules that can regulate it is of significance for potential approaches to combat CVD (Rosenson et al., 2012; Du et al., 2015). ABCA1 is the most important membrane transporter for cholesterol efflux from macrophages (Du et al., 2015). Treatment of THP-1-derived macrophages with falcarindiol increased ABCA1 protein expression (Figure 2). In contrast, the expression level of the other two cholesterol transmembrane transporters, SR-B1 and ABCG1, remained unchanged. In THP-1 macrophages, SR-B1 was shown to be co-expressed with caveolin-1 and to enhance selectively cholesterol ester uptake rather than cholesterol efflux (Matveev et al., 1999). Interestingly, both ABCA1 and ABCG1 proteins seem to be tightly regulated by PPARγ as shown in studies using full agonists of PPARγ (Chawla et al., 2001; Ozasa et al., 2011). Falcarindiol, in contrast, works as a partial PPARγ agonist in HEK-293 cells (Atanasov et al., 2013). Thus, the fact that the abundance of ABCG1 protein did not significantly increase in response to falcarindiol might be explained by the partial agonism of the compound. The contribution of PPARγ to falcarindiol-induced ABCA1 protein expression was demonstrated by co-treatment experiment with BADGE, a PPARγ antagonist (Figure 3). Numerous reports suggest a synergistic effect between ABCA1 and ABCG1 in the context of cholesterol efflux (Gelissen et al., 2006; Hsieh et al., 2014). Although they exhibited a similar degradation rate in CHO cells, ABCG1 degradation seems to work by different degradation mechanisms than that of ABCA1. In addition, there are differences in the protein stability of two ABCG1 isoforms (Gelissen et al., 2010).

We also demonstrate that falcarindiol inhibits protein degradation of ABCA1 in the presence of cycloheximide, a *de novo* protein synthesis inhibitor (Figure 4). Several proteolytic pathways have been described to mediate ABCA1 degradation in different cell types, particularly the calpain-, lysosome-, and proteasome-mediated pathways (Wang et al., 2003; Mizuno et al., 2011; Liu and Tang, 2012; Yokoyama et al., 2012). Applying specific inhibitors of these three proteolytic pathways, we found that falcarindiol mimics the effect of lysosomal proteolysis inhibitor chloroquine on ABCA1 levels (Figure 5).

Cathepsins are well-established lysosomal proteases (Turk et al., 2012). A variety of cathepsins (e.g., cathepsin B, D, K, L, S) are known to be expressed and to serve important regulatory functions in macrophages (Punturieri et al., 2000; Beers et al., 2003; Bracke et al., 2005; Vasiljeva et al., 2006). Measuring the activity of lysosomal cathepsins known to be expressed in macrophages revealed that falcarindiol simultaneously suppresses the activity of cathepsin B, S, and K. Among these three cathepsins, cathepsin S and K were proposed to reduce cholesterol efflux from macrophages (Lindstedt et al., 2003; Lutgens et al., 2007) and knockout of cathepsin S or K genes was shown to lead to decreased atherosclerosis in mice (Sukhova et al., 2003; Lutgens et al., 2006). Cathepsin B was also found to be highly expressed and active in murine atherosclerotic lesions (Chen et al., 2002; Lutgens et al., 2007). The potential of cathepsin B, S, and K to influence ABCA1 protein level regulation further supports the concept for their implication in atherosclerosis. To the best of our knowledge, interference of falcarindiol with cathepsins or with lysosomal function was never reported before.

In summary, our results demonstrate for the first time that falcarindiol is able to increase macrophage cholesterol efflux. Falcarindiol upregulates ABCA1 protein level by promoting its PPARγ-driven gene expression and by extending its protein half-life. Moreover, our findings reveal the inhibition of lysosomal proteases as a new possible mechanism of action of this important dietary constituent. These data advance our knowledge for the molecular mechanisms, by which small molecules regulate macrophage cholesterol efflux, and might have implication for the development of novel therapeutics or dietary supplements for the prevention or treatment of CVD.

## Conclusion

In the present study, we demonstrated that falcarindiol enhances cholesterol efflux and increases ABCA1 protein level by two complementary mechanisms, i.e., promoting ABCA1 gene expression and inhibiting ABCA1 protein degradation.

## Author contributions

LW: Performed most of the experiments, analyzed the results, and drafted the manuscript. VP and NS: Conducted the experiments concerning the expression of transporters. AA and VD: Supervised the study. All authors revised the manuscript.

### Conflict of interest statement

The authors declare that the research was conducted in the absence of any commercial or financial relationships that could be construed as a potential conflict of interest.
